# Comparative Analysis of Muscle Activity and Circulatory Dynamics: A Crossover Study Using Leg Exercise Apparatus and Ergometer

**DOI:** 10.3390/medicina60081260

**Published:** 2024-08-03

**Authors:** Nobuhiro Hirasawa, Yukiyo Shimizu, Ayumu Haginoya, Yuichiro Soma, Gaku Watanabe, Kei Takehara, Kayo Tokeji, Yuki Mataki, Ryota Ishii, Yasushi Hada

**Affiliations:** 1 Graduate School of Comprehensive Human Sciences, University of Tsukuba, Tsukuba 305-8575, Japan; s2130387@u.tsukuba.ac.jp (N.H.);; 2 Department of Rehabilitation Medicine, University of Tsukuba Hospital, Tsukuba 305-8576, Japan; 3 Department of Rehabilitation Medicine, Ibaraki Prefectural University of Health Sciences Hospital, Ami 300-0394, Japan; 4 Department of Rehabilitation Medicine, Institute of Medicine, University of Tsukuba, Tsukuba 305-8575, Japan; 5 Department of Rehabilitation, University of Tsukuba Hospital, Tsukuba 305-8576, Japan; 6 Department of Biostatistics, Institute of Medicine, University of Tsukuba, Tsukuba 305-8575, Japan

**Keywords:** venous thrombosis, blood flow, exercise

## Abstract

*Background and Objectives*: Bedridden patients are at a high risk of venous thromboembolism (VTE). Passive devices such as elastic compression stockings and intermittent pneumatic compression are common. Leg exercise apparatus (LEX) is an active device designed to prevent VTE by effectively contracting the soleus muscle and is therefore expected to be effective in preventing disuse of the lower limbs. However, few studies have been conducted on the kinematic properties of LEX. Therefore, this study aimed to compare the exercise characteristics of LEX with those of an ergometer, which is commonly used as a lower-limb exercise device, and examine its effect on the two domains of muscle activity and circulatory dynamics. *Materials and Methods*: This study used a crossover design in which each participant performed both exercises to evaluate the exercise characteristics of each device. Fifteen healthy adults performed exercises with LEX and an ergometer (Terasu Erugo, SDG Co., Ltd., Tokyo, Japan) for 5 min each and rested for 10 min after each exercise. Muscle activity was measured using surface electromyography (Clinical DTS, Noraxon, Scottsdale, AZ, USA), and circulatory dynamics were recorded using a non-invasive impedance cardiac output meter (Physioflow Enduro, Manatec Biomedical, Paris, France). The primary outcome was the mean percentage of maximum voluntary contraction (%MVC) of the soleus muscle during exercise. *Results*: The mean %MVC of the soleus muscle was significantly higher in the LEX group, whereas no significant differences were observed across the periods and sequences. Heart rate, stroke volume, and cardiac output increased during exercise and decreased thereafter; however, the differences between the devices were not significant. *Conclusions*: LEX may not only have a higher thromboprophylaxis effect, but also a higher effect on preventing muscle atrophy as a lower-extremity exercise device. Additionally, LEX could potentially be used safely in patients who need to be monitored for changes in circulatory dynamics.

## 1. Introduction

Patients who need to stay in bed, such as postoperative and cancer patients, have a high risk of developing venous thromboembolism (VTE), encompassing deep vein thrombosis and pulmonary embolism (PE). PE is a serious disease that is also associated with sudden death. Although the exact number is unknown, it is estimated that about 900,000 people are affected by VTE each year in the United States, and 60,000–100,000 die from this disease [1,2]. Thus, because VTE is a serious complication, preventive measures are essential for these patients. Commonly used methods of VTE prevention include mechanical and pharmacological thromboprophylaxis. However, pharmacological prophylaxis may not be applicable to patients with a risk for bleeding, such as postoperative patients or those with trauma. Thus, mechanical thromboprophylaxis is also useful. Common mechanical devices for preventing VTE include elastic compression stockings and intermittent pneumatic compression. Both of them focus on the blood flow, which is one of the components of Virchow’s triad [2,3,4]. Although these are all passive devices, bed rest may lead to deleterious effects such as disused muscle atrophy in the lower extremity. Indeed, Bamman et al. reported a 15% decrease in maximal knee extension muscle strength after 14 days of bed rest [5]. Conversely, leg exercise apparatus (LEX) is an active device and is expected to compensate for these disadvantages. LEX was originally developed as a novel prophylactic device against VTE by the Department of Rehabilitation Medicine at the University of Tsukuba Hospital. Although early ambulation is equally important as an active mechanical prophylaxis, some patients are unable to walk early owing to dysfunction or rest orders. LEX is beneficial because it may be used in such patients.

**Figure 1:**
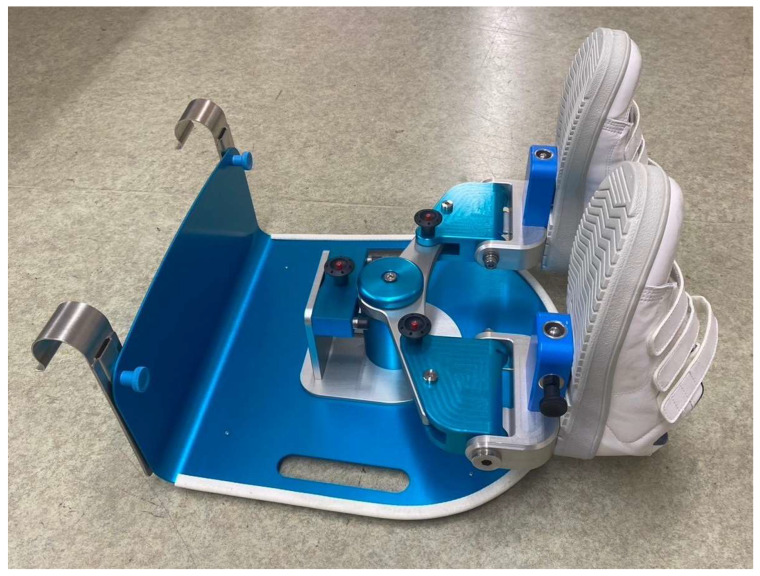
LEX, which comprises right and left pedals, shoes, a metal base, hooks, and a motion control mechanism. LEX: leg exercise apparatus.

LEX consists of pedals for both feet and a motion-control mechanism (Figure 1). The device can be placed on a bed and attached to the bed backboard with hooks, allowing patients to perform leg exercises while lying down. LEX enables various active leg movements, including dorsiflexion, plantar flexion, ankle eversion and inversion, and multi-joint leg movements. Shimizu et al. reported that this combined-motion exercise with LEX improved femoral venous flow volume compared to single-motion exercise of the ankle [6,7]. In addition, LEX is designed to activate the soleus muscle, which is a common site of thrombosis and is associated with the development of fatal pulmonary thromboembolisms [8,9]. As described above, LEX is unique compared with other thromboprophylaxis devices in that it promotes active exercise of the lower extremities. Because of these active mechanisms, LEX is expected to be useful not only as a thromboprophylaxis device, but also as a device to prevent disuse of the lower limbs.

Even in bedridden patients, exercise is useful in preventing body disuse; however, the exercise load should be adjusted according to the patients’ comorbid diseases, such as chronic heart failure. When LEX is applied as an exercise device, it is important to understand its physiological effects, including muscle activity and circulatory dynamics, because physicians and physical therapists generally consider the effects of various types of exercise and recommend appropriate exercise therapy for each patient. However, although LEX’s potential for VTE prophylaxis has been reported, its physiological effects have not been adequately studied [6,8,10,11,12]. Previous evaluations that investigated muscle activity with LEX only compared the effects of exercise with and without LEX and not with other devices [8]. Based on their report, we hypothesized that LEX would promote active muscle contraction in the lower extremities and be useful in preventing their disuse. However, the effectiveness of LEX has not been compared with those of other devices, and further verification is required before LEX can be applied as an exercise device. Regarding circulatory dynamics, only a few studies have compared vital signs, such as pulse rate, blood pressure, and lower-extremity blood flow before and after LEX, but no studies have examined stroke volume or cardiac output.

Past studies have investigated the feasibility and safety of LEX exercise in patients after total joint arthroplasty of the lower extremities and in those with spinal diseases [11,13]. However, for the safe application of this device in patients with stroke or cardiovascular disease, those on dialysis, and other patients who require attention to circulation, it is necessary to understand the extent of changes in the circulatory dynamics. Therefore, we conducted a single-institution crossover study to evaluate the exercise characteristics of LEX in two domains–muscle activity and circulatory dynamics–by comparing it to an ergometer, which is commonly used as a lower-limb exercise device.

## 2. Materials and Methods

### 2.1. Ethical Considerations

This study was conducted in accordance with the principles of the Declaration of Helsinki. Prior to the commencement of the study, approval was obtained from the Institutional Review Board (IRB) of the University of Tsukuba Hospital (R05-091), ensuring that all procedures adhered to rigorous ethical standards. The IRB review included a detailed assessment of the study’s objectives, methods, potential risks and benefits, participant privacy protection, and informed-consent process. Written informed consent was obtained from all the participants before their inclusion in the study. Participants were informed about the nature of the study, potential risks and benefits, and their right to withdraw from the study at any time without penalty. The informed-consent process ensured that participants had ample opportunity to ask questions and receive comprehensive explanations before agreeing to participate in this study.

### 2.2. Participants

We recruited volunteers in this study; the eligible participants were healthy adults aged between 20 and 60 years. To eliminate the possibility that the exercise could not be performed properly and safely, several exclusion criteria were established: a history of fractures or surgery of the lower extremities, thrombosis, or respiratory or cardiovascular disease. The required number of participants was calculated based on a previous study that compared the differences in lower-extremity motion with and without LEX. The mean and standard deviation of the difference in the mean percentage of maximum voluntary contraction (%MVC) of the soleus muscle with LEX and without LEX were assumed to be 12.6 and 17.3, respectively. Based on a Student’s *t*-test with a significance level of 0.05 and power of 0.80, 15 participants were recruited [12].

### 2.3. Exercise Protocols and Measurements

**Figure 2:**
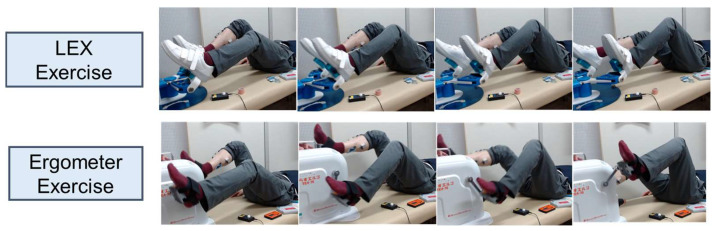
Illustrations depicting each exercise. The **upper** photos show the exercise with LEX and the **lower** photos show the exercise with an ergometer.

This study used a crossover design in which each participant performed both exercises to evaluate the exercise characteristics of each device. Each participant was instructed on how to perform the exercises and asked to lie down in a supine position on the examination bed for 10 min. They then performed LEX and ergometer (Terasu Erugo, SDG Co., Ltd., Japan) exercises for 5 min each in the supine position, followed by a 10 min rest period after each exercise session. Shimizu et al. conducted a future exercise with LEX four times a day for 5 min each time [6]. In this study, as in previous studies, the exercise duration was set to 5 min, assuming its application to actual clinical settings [11,13]. The order of exercises was randomly assigned to each participant using block randomization. The interval between each exercise was set at 10 min or longer to reduce carryover effects but so as not to force excessive rest in healthy participants. Both exercises were performed using a metronome set at 30 cycles/min. The load of the variable-load ergometer was set at 20 watts, the lowest of the devices used in this study, in anticipation of future application to a wide range of patients, including those with low exercise tolerance. The joint angles of the lower extremities during each exercise were not strictly controlled, allowing the participants to perform each exercise comfortably. These exercises are illustrated in Figure 2.

Muscle activity was recorded using wireless surface electromyography (EMG) (Clinical DTS, Noraxon, USA) in the right tensor fasciae latae (TFL), vastus lateralis (VL), biceps femoris (BF), lateral head of the gastrocnemius (GC), soleus (SOL), and tibialis anterior (TA) muscles. Pairs of Ag-AgCl surface electrodes (34 mm in diameter) were applied at a distance of 30 mm between the electrode centers. The electrodes were placed according to the recommended sites using Surface EMG for Non-Invasive Assessment of Muscles after the skin was thoroughly wiped clean. To record the knee joint movements, motion sensors (Noraxon Ultium Motion, Noraxon, USA) were attached to the participants’ right thighs and shanks. The motion and EMG measurements were synchronized with video recordings using a USB camera (C920 HD PRO Web camera; Logicool, Tokyo, Japan). The sampling frequencies were 1500 Hz for the surface electromyograph and 200 Hz for the motion analyzer. These signals were filtered at 10–500 Hz to remove movement artifacts and high-frequency noise using MyoResearch 3.0 (version 3.18; Noraxon, USA). EMG signals were amplified using a gain factor of 500. The filtered and amplified EMG signals were rectified and smoothed using a root mean square (RMS) algorithm with a 50 ms window. The maximum voluntary contraction (MVC) of each muscle was measured using manual resistance after both exercise tests were completed. The participants were instructed to exert maximum effort for 5 s. The MVC data were smoothed using the RMS algorithm with a 50 ms window, and the amplitude was normalized to the peak value using a smoothing window of 1000 ms. The amplitude of the EMG signals during exercise was normalized to the MVC, and the data were expressed as %MVC. One cycle of each exercise was defined as the interval from maximum right knee extension to the subsequent maximum right knee extension. Among the waveforms averaged over all properly recorded cycles, the maximum and mean values were defined as the peak %MVC and mean %MVC, respectively, and used for the analysis.

Circulatory dynamics were recorded using an impedance cardiac output meter (PhysioFlow Enduro, Manatec Biomedical, France). Using an impedance meter, we can non-invasively evaluate circulatory dynamics, unlike conventional cardiac output measurement methods that rely on the Fick principle. This makes the impedance meter particularly suitable for assessing exercise characteristics in healthy participants. Additionally, because the impedance meter provided results that correlated well with those obtained using invasive methods, it was selected for this study [14,15]. Measurements were acquired at the beginning of exercise, at 2.5 min into exercise, at the end of exercise, and at 1, 5, and 10 min after the start of the rest period, using the median value of the preceding 1 min interval at each time point.

**Figure 3:**
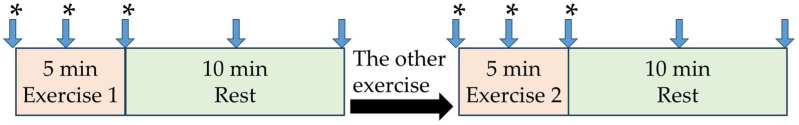
Schematic diagram of the exercises. “Exercise 1” indicates the first exercise performed, and “Exercise 2” indicates the second exercise performed. The arrows indicate the time point at which the circulatory dynamics were assessed, and the asterisks (*) indicate the time point at which the modified Borg scale score was obtained.

Subjective fatigue was assessed using the modified Borg Scale scores separately for respiration and lower extremities at each of the following time points: immediately before exercise, 2.5 min after the start of exercise, and at the end of exercise [16]. These measurement points and the flow of the exercises are described in Figure 3.

### 2.4. Outcome Measures

The primary outcome of this study was the mean %MVC of the soleus muscle during exercise, and the secondary outcomes were the mean %MVC of the other muscles (tensor fascia latae, biceps femoris, vastus lateralis, lateral head of the gastrocnemius, and tibialis anterior), peak %MVC of each muscle, cardiac output, stroke volume, heart rate, and modified Borg scale.

### 2.5. Statistical Analysis

To assess the impact of carryover and period effects in the crossover trial, statistical analyses were conducted for the %MVC of each muscle using a linear mixed-effects model with type, period, and sequence of exercise as fixed effects and participant as a random effect. Statistical significance was set at *p* < 0.05. All analyses were performed using R version 4.3.1.

## 3. Results

Fifteen participants (nine men and six women) were included in this study. The age range of the participants was 26–59 years, and the average age of the participants was 35.7 years with a standard deviation of 10.8 years. Eight participants performed the LEX exercise first, and seven the ergometer exercises first. The characteristics and order of the exercises for each participant are listed in Table 1.

All mean and peak %MVC values obtained in this study are shown in Table 2, and the results of the statistical analysis using a linear mixed-effects model are shown in Table 3 and Table 4. No significant differences according to period or sequence were observed in the mean and peak MVC results for either muscle type. When comparing the mean %MVC of the soleus muscle during exercise, which was the primary outcome measure, the LEX group showed significantly higher values than the ergometer group, and the ratio between the LEX and ergometer group values was 1.8 (*p* = 0.032). Unlike the mean %MVC, there was no significant difference between the groups in the peak %MVC of the soleus muscle, although there was a trend toward higher values in the LEX group, with a ratio of 1.5 (*p* = 0.355). However, the LEX group had significantly higher values in the gastrocnemius muscle, with a ratio of 3.5 (*p* < 0.001). The mean and peak %MVC of the tensor fascia latae and tibialis anterior muscles were significantly higher in the LEX group, with the ratios in the mean %MVC for the tensor fascia latae and tibialis anterior muscles, and the peak %MVCs for them were 2.0, 3.5, 1.7, and 4.5, respectively (*p* = 0.012, 0.002, 0.013, 0.006, respectively). In the other muscles, there were no statistically significant differences between the LEX and ergometer groups in either the mean %MVC or peak %MVC.

**Figure 4:**
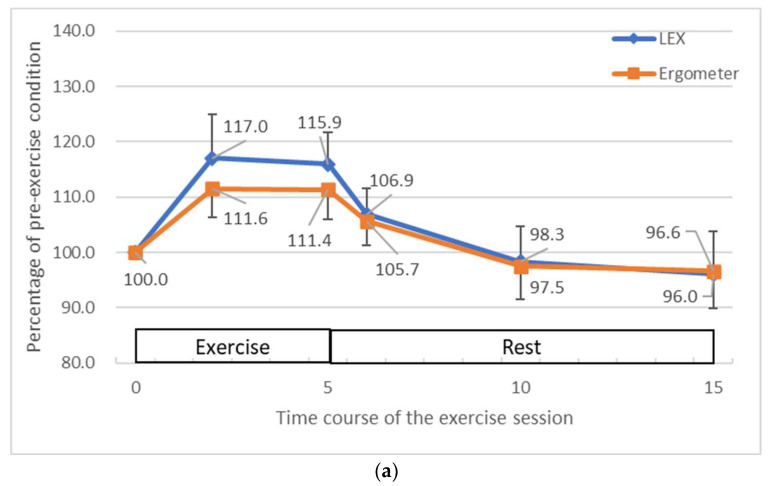

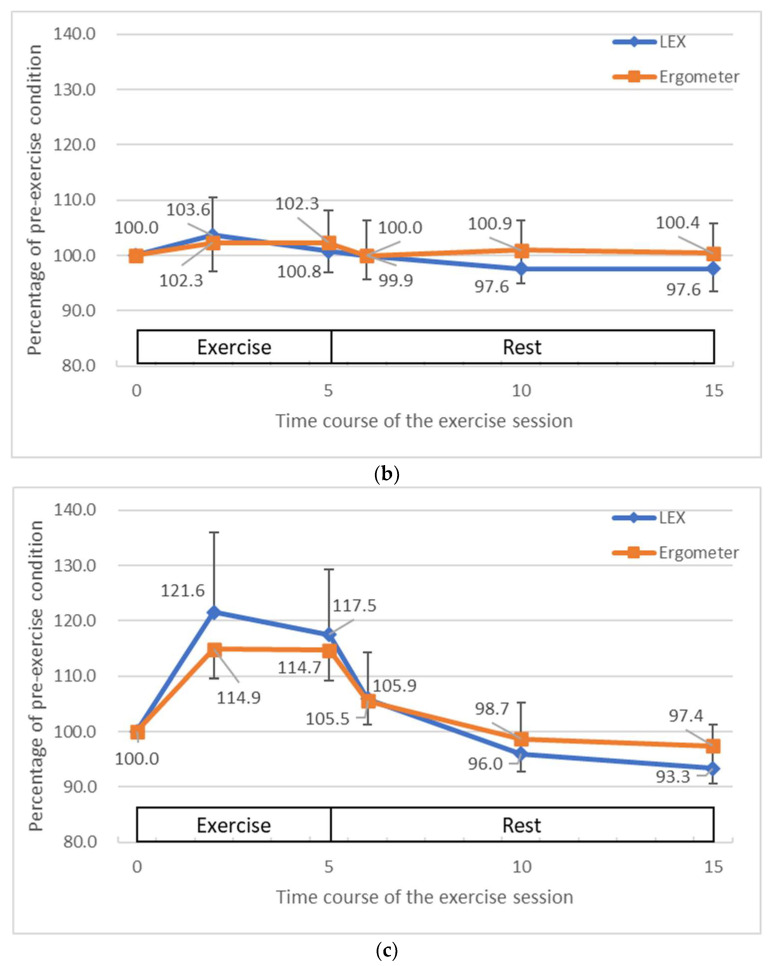
The average changes in heart rate (**a**), stroke volume (**b**) and cardiac output (**c**) during and after exercises with LEX and the ergometer. The vertical axis represents the percentage of the value at rest and the horizontal axis the number of minutes elapsed since the start of the exercise session. Exercise was performed for 5 min after the start of the session and rest was performed thereafter. LEX: leg exercise apparatus.

Compared to the resting state before exercise, heart rate, stroke volume, and cardiac output were increased during exercise (Figure 4). The maximum increase in heart rate was 17.0% for LEX and 11.6% for the ergometer; the maximum increase in stroke volume was 3.6% for LEX and 2.3% for the ergometer; and the maximum increase in cardiac output was 21.6% for LEX and 14.9% for the ergometer. All these values were decreased to near baseline levels after the end of the exercise. There were no apparent differences in circulatory changes between the exercise types.

**Figure 5:**
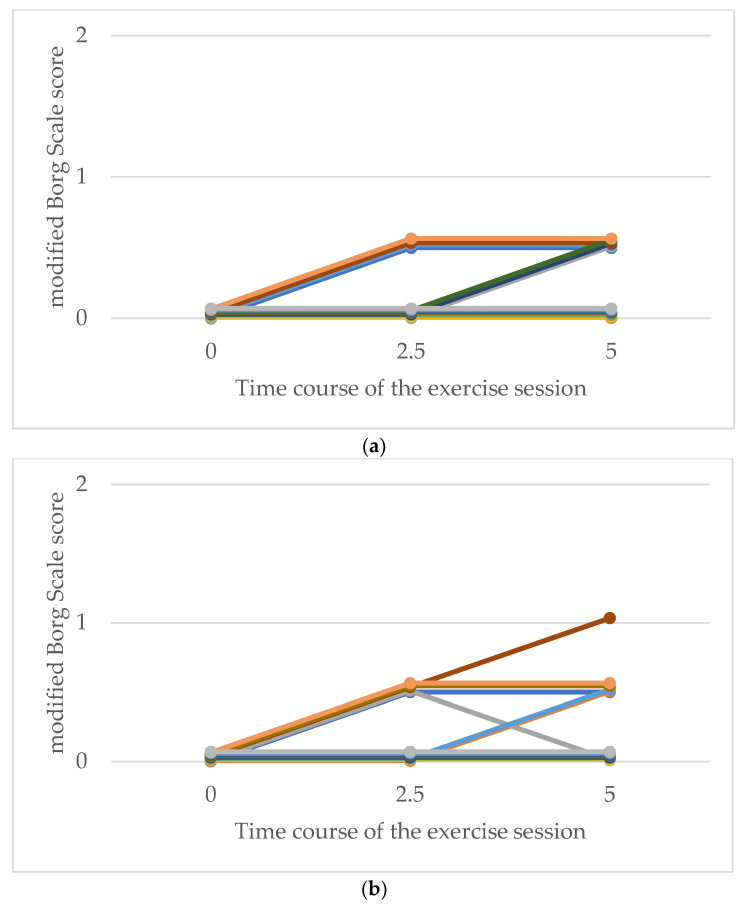
Comparative modified Borg scale ratings for respiration following exercise with LEX (**a**) and ergometer (**b**). The vertical axis represents the modified Borg scale score for respiration in each participant, and the horizontal axis represents the number of minutes elapsed since the start of the exercise session. LEX: leg exercise apparatus.

**Figure 6:**
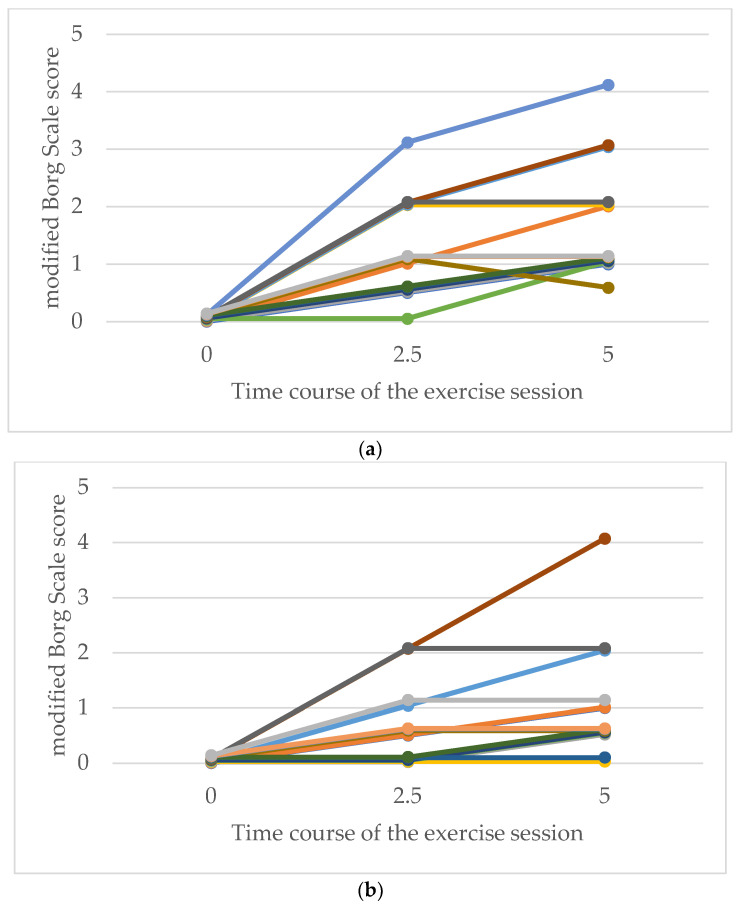
Comparative modified Borg scale ratings for leg fatigue following exercise with LEX (**a**) and the ergometer (**b**). The vertical axis represents the modified Borg scale score for leg fatigue in each participant, and the horizontal axis represents the number of minutes elapsed since the start of the exercise session. LEX: leg exercise apparatus.

With respect to the modified Borg scale scores for respiratory fatigue, more participants in both groups reported higher levels of fatigue as the exercise time progressed. Owing to the small number of participants in this study, no statistical analysis was performed on the modified Borg scale scores among the secondary endpoints. However, there was no clear difference in perceived respiratory fatigue between the exercise types, and all ratings remained low, ranging from 0 (no breathlessness) to 1 (very slight breathlessness) (Figure 5). Regarding leg fatigue, more participants in both groups reported higher fatigue as the exercise time progressed. Although the highest leg fatigue score was 4 (somewhat severe) in the LEX and ergometer groups, more participants in the LEX group tended to report higher levels of leg fatigue (Figure 6).

## 4. Discussion

In the present study, differences in exercise characteristics with LEX and those with an ergometer were evaluated in terms of muscle activity and changes in circulatory dynamics. Because this was a crossover study, the period and sequence of exercises were also analyzed to eliminate factors that could affect results other than the type of exercise; however, these factors did not show significant differences in any of the muscles, suggesting that the carryover and period effects in the crossover study were eliminated.

The mean %MVC of the soleus muscle, the primary outcome, was significantly higher in the LEX group. LEX is designed to stimulate the contraction of the soleus muscle, and Tanaka et al. reported that integrated electromyograms of the soleus muscle during exercise were generally higher when using LEX compared to that when not using it [8]. Our study also revealed that soleus muscle activity measured using LEX was higher than that measured using an ergometer. One advantage of LEX is its potential usefulness as a thrombogenic device, as suggested in multiple studies [6,7,8,10,11,12]. Although no studies have directly compared the thrombogenic rates between ergometers and LEX, this difference in soleus muscle activity indicates that LEX may be more effective in preventing thrombosis by promoting muscle activation in an important area related to venous return.

Regarding lower-limb exercise devices, an ergometer is commonly used in the rehabilitation field as an exercise device for the lower limbs and has the advantage of being suitable for use in bed. In recent years, dialysis patients have been increasingly exercising while lying in bed during hemodialysis, with the aim of preventing the loss of motor endurance; an ergometer is useful in such situations [15]. However, our study suggests that the LEX may be a more effective lower-extremity exercise device for use in bed in terms of its exercise effect on the soleus muscle.

The peak %MVC of the soleus tended to be higher in the LEX group, although the difference was not significant. However, the peak %MVC of the gastrocnemius muscle was significantly higher in the LEX group. Previous research by Hébert-Losier et al. showed that the gastrocnemius, a biarticular muscle, exhibited muscle activity measured by surface EMG, which is related to the knee joint flexion angle, with higher activity in the knee joint extension position than in the flexion position [17]. Although this study did not strictly define the knee angle, which allowed participants to exercise in a comfortable position, LEX might involve more knee extension during ankle plantar flexion than the ergometer. This could explain the difference in the peak values observed during one exercise cycle. To date, no comparative studies on the changes in knee and ankle joint angles during one cycle of LEX exercise have been conducted, highlighting the need for future research. For other muscles, the mean and peak %MVC of the tensor fasciae latae and tibialis anterior tended to be higher during the LEX exercise. The tensor fascia latae is marginally involved in hip and knee flexion, whereas the tibialis anterior is primarily involved in ankle dorsiflexion. LEX, by design, involves ankle plantar flexion during lower-extremity extension and dorsiflexion during flexion. This likely requires more effort to pull the lower limbs back, involving hip flexion, knee flexion, and ankle dorsiflexion, compared to the ergometer. This increased effort may explain why the LEX group reported higher levels of lower-extremity fatigue on the modified Borg scale scores. However, leg fatigue with LEX exercises is only approximately one level higher than that with ergometer exercises, and, as with ergometers, LEX could be used in a wide range of patients. Although the ergometer load was set at 20 watts in this study, it is undeniable that a higher load could result in higher activity in each muscle than that when using LEX. However, it is assumed that this would result in higher perceived fatigue. When performed as a low-intensity exercise, as in this study, the advantage of LEX is that it can stimulate triceps surae muscle activity more efficiently than the ergometer.

This study also investigated the effects of each exercise on circulatory dynamics. In general, immediately after the start of exercise, the cardiac output increases to meet the higher metabolic demands of the active muscles, mainly owing to the suppression of parasympathetic activity. This increase is owing to an increase in heart rate and stroke volume owing to changes in autonomic nervous system activity, increased venous return, and increased ventricular filling in accordance with the Frank–Starling law [18]. In this study, we observed that heart rate, stroke volume, and cardiac output were increased after the start of exercise and decreased after the end of exercise. However, none of these measures were increased further at 5 min compared with 2.5 min after the start of exercise, suggesting that they reached a plateau earlier. This was likely owing to the low intensity of LEX and ergometer exercises, in which the maximum increase in heart rate was only about 17%, and it can be said that there were no clinically significant differences between the two exercise types. From a circulatory dynamic standpoint, although it has been validated with low-intensity and short-duration exercise, the results suggest that LEX, like an ergometer, could potentially be used as a lower-extremity exercise device for patients needing to monitor circulatory dynamics, such as dialysis patients. Further research is required to determine the safety of prolonged exercise using LEX.

This study is particularly valuable because it is the first to compare the exercise characteristics of LEX with those of another device. Compared to ergometers, which are commonly used for lower-limb exercises, LEX was more effective in eliciting activity in the soleus muscle, a common site of thrombus formation, thereby demonstrating its usefulness as a lower-limb exercise device. Changes in cardiac performance as a secondary outcome have not been previously reported, adding a unique aspect to this study.

Despite these advantages, this study had several limitations. First, there were large differences in the surface EMG results among participants, including some outliers. Even in actual clinical practice, it is not easy to strictly instruct patients on the angles of each joint and the movements of the lower limbs. Therefore, we did not define these movements strictly in this study either, which may be the reason for this variability. Additionally, both exercises were performed at low loads and speeds to make them accessible to bedridden patients. Therefore, it can be said that the results of this study reflect its use in clinical situations; nevertheless, a more rigorous study design might have yielded different results.

Second, the participants in this study were healthy individuals only. Although we enrolled them to match the background of each participant as closely as possible, if LEX is used for patients with impaired lower-limb movement owing to hemiplegia or other reasons, it is possible that similar muscle activity may not be obtained or that the patient may not even be able to perform the proper movements. However, it is assumed that these people are more prone to VTE owing to immobilization of the disabled limb and they are the ones who need more thromboprophylaxis.

Third, although significant differences were found in the primary outcomes, the small sample size (15 participants) may not have been sufficient for robust comparisons of secondary outcomes. Additionally, statistical comparisons were not performed for circulatory dynamics and fatigue owing to the small sample size, which is another limitation. Further studies with larger sample sizes and more controlled conditions are required to confirm these findings and provide more comprehensive insights.

Building on the current study’s findings, several avenues for future research can be identified. Because the present study suggested that there was no marked difference in the effect on circulatory dynamics in the use of the LEX compared to that of the ergometer, a feasibility study is being considered for application in patients with cerebrovascular disease and those on hemodialysis. Furthermore, in this study, the exercise duration was set to 5 min based on previous studies, but it is assumed that the exercise duration will not be limited to 5 min when LEX is used as a lower-limb exercise device in clinical situations. Therefore, a comparative study with a greater variety of loading durations will provide a more accurate understanding of the effects of LEX on circulatory dynamics. In addition, further investigation is required not only of short-term outcomes, such as changes in muscle activity and blood flow, but also of long-term outcomes such as activities of daily living, quality of life, incidence of VTE, and length of hospital stay. Such investigations will help characterize LEX exercise and improve exercise prescriptions for different populations.

## 5. Conclusions

This was the first trial to compare the exercise characteristics of LEX with those of another device. This study revealed that the mean %MVC of the soleus muscle was significantly higher in the LEX group than in ergometer group. This suggests that LEX may have not only a higher thromboprophylaxis effect but also a higher effect of preventing muscle atrophy as a lower-extremity exercise device. Additionally, this is the first report to compare the effects of LEX exercises on circulatory dynamics with those of other devices. Although the number of participants was small and no statical analysis was performed on the assessment of circulatory dynamics and fatigue, there was no apparent difference in the effect of LEX on them compared to the ergometer. This indicates that LEX can be safely used as an ergometer in terms of its effects on the circulatory system. These findings suggest that the device could be applied not only as a thromboprophylaxis device but also as a lower-extremity exercise device for patients who need to be monitored for circulatory changes in the future. Since this study was conducted on healthy participants, further research is needed to determine whether similar results can be obtained in people with health issues.

## Figures and Tables

**Table 1:** Patients enrolled in this study.

Participant	Sex	Age (years)	First Exercise	Second Exercise
1	Male	32	LEX	Ergometer
2	Female	47	Ergometer	LEX
3	Female	26	Ergometer	LEX
4	Male	29	LEX	Ergometer
5	Male	23	LEX	Ergometer
6	Male	32	Ergometer	LEX
7	Male	23	Ergometer	LEX
8	Female	36	LEX	Ergometer
9	Female	32	LEX	Ergometer
10	Female	26	Ergometer	LEX
11	Female	40	LEX	Ergometer
12	Male	57	Ergometer	LEX
13	Male	38	LEX	Ergometer
14	Male	35	Ergometer	LEX
15	Male	59	LEX	Ergometer

LEX: leg exercise apparatus.

**Table 2:** Mean and peak %MVC of each muscle in LEX group and ergometer group.

(**a**) Mean %MVC of each muscle			
	**LEX**	**Ergometer**	**LEX/Ergometer**
TFL	6.0 ± 4.9	4.9 ± 3.0	2.0
VL	6.6 ± 7.4	7.4 ± 6.6	1.0
BF	10.2 ± 11.9	11.9 ± 9.0	1.1
GC	14.9 ± 6.0	6.0 ± 5.8	2.6
SOL	18.2 ± 19.9	19.9 ± 10.3	1.8
TA	6.5 ± 4.6	4.6 ± 1.9	3.5
(**b**) Peak %MVC of each muscle			
	**LEX**	**Ergometer**	**LEX/Ergometer**
TFL	9.5 ± 7.1	7.1 ± 5.7	1.7
VL	10.6 ± 10.0	10.0 ± 13.3	0.8
BF	16.2 ± 18.0	18.0 ± 17.7	0.9
GC	39.0 ± 20.0	20.0 ± 11.1	3.5
SOL	37.5 ± 43.8	43.8 ± 25.3	1.5
TA	15.1 ± 13.6	13.6 ± 3.4	4.5

%MVC: percentage of maximum voluntary contraction; LEX: leg exercise apparatus.

**Table 3:** Mean %MVC of each muscle.

(**a**) Tensor fasciae latae			
	**Regression Coefficient**	**Standard Error**	***p*** **-Value**
Type (reference: ergometer)	2.946	1.013	0.012
Period (reference: 1)	−0.595	1.013	0.567
Sequence (reference: 1)	0.007	1.757	0.997
(**b**) Vastus lateralis			
	**Regression Coefficient**	**Standard Error**	***p*** **-Value**
Type (reference: ergometer)	−0.127	1.308	0.924
Period (reference: 1)	−2.637	1.308	0.065
Sequence (reference: 1)	4.382	4.160	0.311
(**c**) Biceps femoris			
	**Regression Coefficient**	**Standard Error**	***p*** **-Value**
Type (reference: ergometer)	1.164	0.645	0.094
Period (reference: 1)	0.454	0.645	0.494
Sequence (reference: 1)	1.348	6.075	0.828
(**d**) Gastrocnemius			
	**Regression Coefficient**	**Standard Error**	***p*** **-Value**
Type (reference: ergometer)	9.107	1.466	<0.001
Period (reference: 1)	0.155	1.466	0.918
Sequence (reference: 1)	2.834	2.257	0.231
(**e**) Soleus			
	**Regression Coefficient**	**Standard Error**	***p*** **-Value**
Type (reference: ergometer)	7.760	3.228	0.032
Period (reference: 1)	−1.858	3.228	0.575
Sequence (reference: 1)	−7.915	7.789	0.328
(**f**) Tibialis anterior			
	**Regression Coefficient**	**Standard Error**	***p*** **-Value**
Type (reference: ergometer)	4.580	1.327	0.002
Period (reference: 1)	−0.836	1.327	0.534
Sequence (reference: 1)	−0.331	1.327	0.805

%MVC: percentage of maximum voluntary contraction; LEX: leg exercise apparatus.

**Table 4:** Peak %MVC of each muscle.

(**a**) Tensor fasciae latae			
	**Regression Coefficient**	**Standard Error**	***p*** **-Value**
Type (reference: ergometer)	3.708	1.294	0.013
Period (reference: 1)	−0.478	1.294	0.718
Sequence (reference: 1)	1.036	2.841	0.721
(**b**) Vastus lateralis			
	**Regression Coefficient**	**Standard Error**	***p*** **-Value**
Type (reference: ergometer)	−3.071	2.713	0.278
Period (reference: 1)	−5.780	2.713	0.053
Sequence (reference: 1)	8.609	7.124	0.248
(**c**) Biceps femoris			
	**Regression Coefficient**	**Standard Error**	***p*** **-Value**
Type (reference: ergometer)	−1.363	1.635	0.420
Period (reference: 1)	2.187	1.635	0.204
Sequence (reference: 1)	5.330	10.766	0.629
(**d**) Gastrocnemius			
	**Regression Coefficient**	**Standard Error**	***p*** **-Value**
Type (reference: ergometer)	28.253	5.150	<0.001
Period (reference: 1)	5.465	5.150	0.308
Sequence (reference: 1)	11.317	5.764	0.071
(**e**) Soleus			
	**Regression Coefficient**	**Standard Error**	***p*** **-Value**
Type (reference: ergometer)	12.301	12.814	0.355
Period (reference: 1)	1.044	12.814	0.936
Sequence (reference: 1)	−20.265	18.166	0.285
(**f**) Tibialis anterior			
	**Regression Coefficient**	**Standard Error**	***p*** **-Value**
Type (reference: ergometer)	11.611	3.846	0.006
Period (reference: 1)	−1.239	3.846	0.750
Sequence (reference: 1)	−0.051	3.846	0.990

%MVC: percentage of maximum voluntary contraction; LEX: leg exercise apparatus.

## Data Availability

The original contributions presented in this study are included in the article; further inquiries can be directed to the corresponding author.
