# Correction: Hirasawa et al. Comparative Analysis of Muscle Activity and Circulatory Dynamics: A Crossover Study Using Leg Exercise Apparatus and Ergometer. *Medicina* 2024, *60*, 1260

**DOI:** 10.3390/medicina60121958

**Published:** 2024-11-28

**Authors:** Nobuhiro Hirasawa, Yukiyo Shimizu, Ayumu Haginoya, Yuichiro Soma, Gaku Watanabe, Kei Takehara, Kayo Tokeji, Yuki Mataki, Ryota Ishii, Yasushi Hada

**Affiliations:** 1 Graduate School of Comprehensive Human Sciences, University of Tsukuba, Tsukuba 305-8575, Japan; s2130387@u.tsukuba.ac.jp (N.H.);; 2 Department of Rehabilitation Medicine, University of Tsukuba Hospital, Tsukuba 305-8576, Japan; 3 Department of Rehabilitation Medicine, Ibaraki Prefectural University of Health Sciences Hospital, Ami 300-0394, Japan; 4 Department of Rehabilitation Medicine, Institute of Medicine, University of Tsukuba, Tsukuba 305-8575, Japan; 5 Department of Rehabilitation, University of Tsukuba Hospital, Tsukuba 305-8576, Japan; 6 Department of Biostatistics, Institute of Medicine, University of Tsukuba, Tsukuba 305-8575, Japan

## Error in Table

In the original publication [1], the values for the Ergometer Group in “Table 2” were incorrect. Specifically, the standard deviation for the Ergometer Group was mistakenly recorded as the mean value for the Ergometer Group, while the mean value for the Ergometer Group was replaced with the standard deviation for the LEX Group. This is evident from the fact that the standard deviation for the LEX Group and the mean value for the Ergometer Group are identical, which should not be the case.

Specifically, for “(a) Mean %MVC of each muscle”, the reported values were as follows: TFL at 4.9 ± 3.0, VL at 7.4 ± 6.6, BF at 11.9 ± 9.0, GC at 6.0 ± 5.8, SOL at 19.9 ± 10.3, and TA at 4.6 ± 1.9. The corrected values for this category are as follows: TFL at 3.0 ± 1.7, VL at 6.6 ± 9.0, BF at 9.0 ± 10.1, GC at 5.8 ± 3.9, SOL at 10.3 ± 9.9, and TA at 1.9 ± 1.4.

For “(b) Peak %MVC of each muscle”, the original incorrect values were as follows: TFL at 7.1 ± 5.7, VL at 10.0 ± 13.3, BF at 18.0 ± 17.7, GC at 20.0 ± 11.1, SOL at 43.8 ± 25.3, and TA at 13.6 ± 3.4. The correct values for this category are as follows: TFL at 5.7 ± 3.7, VL at 13.3 ± 18.2, BF at 17.7 ± 21.4, GC at 11.1 ± 8.1, SOL at 25.3 ± 38.5, and TA at 3.4 ± 2.5.

The revised Table 2 is presented below.

**Table 2:** Mean and peak %MVC of each muscle in LEX Group and Ergometer Group.

(**a**) Mean %MVC of each muscle			
	LEX	Ergometer	LEX/Ergometer
TFL	6.0 ± 4.9	3.0 ± 1.7	2.0
VL	6.6 ± 7.4	6.6 ± 9.0	1.0
BF	10.2 ± 11.9	9.0 ± 10.1	1.1
GC	14.9 ± 6.0	5.8 ± 3.9	2.6
SOL	18.2 ± 19.9	10.3 ± 9.9	1.8
TA	6.5 ± 4.6	1.9 ± 1.4	3.5
(**b**) Peak %MVC of each muscle			
	LEX	Ergometer	LEX/Ergometer
TFL	9.5 ± 7.1	5.7 ± 3.7	1.7
VL	10.6 ± 10.0	13.3 ± 18.2	0.8
BF	16.2 ± 18.0	17.7 ± 21.4	0.9
GC	39.0 ± 20.0	11.1 ± 8.1	3.5
SOL	37.5 ± 43.8	25.3 ± 38.5	1.5
TA	15.1 ± 13.6	3.4 ± 2.5	4.5

%MVC: percentage of maximum voluntary contraction; LEX: leg exercise apparatus.

The authors confirm that this typographical error does not impact the core findings or interpretations in the manuscript. All analyses and discussions were based on the correct figures, and this error affects only the presentation of data in the table.

This correction was approved by the Academic Editor. The original publication has also been updated.
